# Observations on Koch’s Lymph

**Published:** 1891-06

**Authors:** Joseph Jones

**Affiliations:** New Orleans, Louisiana


### Original Communications.

### OBSERVATIONS ON KOCH'S LYMPH.

BY JOSEPH JONES, M. D., OF NEW ORLEANS, LOUISIANA.

156 WASHINGTON AVENUE, CORNER OF CAMP STREET,
NEW ORLEANS, LOUISIANA, April 28th, 1891.

*His Excellency, Benjamin Harrison, President of the United States of America, Washington, D. C.:*

SIR—I have the honor to acknowledge the following:

EXECUTIVE MANSION, WASHINGTON, January 19th, 1891.

*Professor Joseph Jones, Charity Hospital, New Orleans, La.:*

MY DEAR SIR—At the President's direction, I beg leave to send you by express one vial of Koch's lymph for such use as you may deem wise to make of it.

It was forwarded to the President by the American minister in Germany.

Very truly yours,

E. W. HALFORD,
Private Secretary.

I beg leave, respectfully, to submit to your Excellency the following brief report on this vial of Koch's lymph :

The small vial of "Koch's Lymph," containing about five grammes (about seventy-six drops) of a dark brownish red liquid, accompanied by directions for its use, signed by Dr. A. Libbertz, of Berlin, Germany, was delivered to me in person, at my office, 36 University Place, by the express agent.

Holding that your Excellency designed this humane bequest, not for private ends, but for the benefit of suffering humanity, and the promotion of scientific inquiry, I placed a portion of "Koch's lymph," contained in the small vial, at the disposal of the medical and surgical staff of the Charity Hospital of Louisiana, as will be seen from the following correspondence:

OFFICIAL BUSINESS.

36 UNIVERSITY PLACE, NEW ORLEANS, LOUISIANA,
January 22d, 1891.

*Professor A. B. Miles, M. D., Resident Surgeon Charity Hospital, New Orleans, La.:*

MY DEAR DOCTOR—On the 22d instant I received by express a small vial of "Koch's Lymph," together with the enclosed communication from the Private Secretary of his Excellency, President Harrison. * * *

I respectfully tender to the Surgeon in charge of the Charity Hospital, and through him to the medical and surgical staff, a portion of the "lymph" for the treatment of the patients in the wards of the Charity Hospital, provided that I be furnished with accurate reports of each and every case thus treated.

Respectfully your obedient servant,

JOSEPH JONES, M. D.,
Visiting Physician Charity Hospital.

CHARITY HOSPITAL, STATE OF LOUISIANA,
NEW ORLEANS, January 26th, 1891.

*Prof. Joseph Jones, M. D., Visiting Physician Charity Hospital. :*

MY DEAR DOCTOR—I beg to acknowledge receipt of your favor of the 22d instant, tendering to the Surgeon in charge of the Charity Hospital, and through him to the medical and surgical staff, a portion of the lymph which you have received from President Harrison.

Accept my thanks for your courtesy in this matter.

I will inform the members of the medical and surgical staff of your kind offer, and refer to you those who desire to experiment with the lymph in their ward service. Very truly yours,

A. B. MILES,
House Surgeon.

We extract the following from the official proceedings of the Board of Administrators of the Charity Hospital, April, 1891.

" Dr. Miles reported relative to ' Koch's lymph,' in which he said the world was taking interest.

" Dr. Joseph Jones had received a vial, and tendered it to the hospital. He had placed a notice on the bulletin board, inviting others to use it in safe bounds, if they thought proper.

" No one had applied to use it. For himself, he did not care to use it yet, as he did not deem the lymph or its substance sufficiently understood.

" It may yet be used in the hospital, but it would be best to await further results from it."

Assisted by my chiefs of clinic, Dr. Stanhope Jones and Dr. J. M. Elliott, I examined the cases in the wards, under my care in the Charity Hospital daily, up to the middle of March, with a view to the use of "Koch's lymph," in the diagnosis and treatment of phthisis pulmonalis and other forms of tubercular disease.

That this agent or drug was not used in the treatment of diseases under my care, in the wards of the Charity Hospital of New Orleans, was due to the following causes:

(*a*) No case presented itself which I deemed suited to the applicatian of " Koch's treatment " without danger to the welfare of the patients.

(*b*) No case presented itself of which the diagnosis was so obscure as to require the institution of a doubtful experiment.

(*c*) Without exception, the patients under my treatment and care in those wards of the Charity Hospital declined to submit to this mode of treatment.

(*d*) The extensive prevalence of *influenza* in a severe and often fatal form, and which attacked with especial violence those suffering with phthisis pulmonalis, rendered the injection of an irritating agent into the living human body hazardous.

In accordance with what I concede to be the humane and charitable intention of your Excellency, I have held the small "vial of Koch's lymph" sacred to scientific and charitable investigations.

I have received a number of applications from physicians and private individuals for the use of this "Koch's lymph," in private practice and in institutions other than the Charity Hospital of Louisiana, and I have uniformly refused such applications. Such applications appear to have been based upon a misapprehension of the intention of your Excellency, and upon ignorance of the therapeutic value and power of a quantity of liquid too small to supply more than one drop and a half to each one of the 52 wards of the Charity Hospital, with a daily average of 550 and an annual average of about 7,000 cases of all diseases.

OUTLINE OF RESULTS OF CHEMICAL AND MICROSCOPICAL EXAMINATION OF THE CONTENTS OF A VIAL OF KOCH'S LYMPH.

The objectives employed in the following observations ranged from $\frac{1}{5}$ to $\frac{1}{15}$ of an inch. Due precautions were taken to secure such results as were possible in the chemical and microscopical manipulation of the small amounts of material.

PROPERTIES OF KOCH'S LYMPH.

1. Reddish-brown liquid, with oily movement and consistency of thin glycerine.
2. Clear, with a few minute flocculi.
3. Musty odor like that of stale beef extract.
4. When burned emitted an odor like burning beef extract.
5. Reaction strongly alkaline.

6. When a drop of the undiluted liquid was placed in the eye of a living animal, it appeared to cause a disagreeable sensation, attended with closing the lids temporarily, but it induced no permanent irritation or inflammation. A repetition of this experiment caused no perceptible injury to the eye or animal.

7. No appreciable effects were induced by the lymph when administered internally, by the mouth, to living animals. The fluid in its innocuous effects, when applied to living mucous membranes, differed from the poisonous alkaloids, and from prussic acid and the cyanides.

8. Mingles rapidly and freely in all proportions with distilled water.

9. When injected in varying degrees of dilution with distilled water (50 per cent., 25 per cent., 10 per cent., 1 per cent., 0.1 per cent.) into the subcutaneous tissues of living animals, cats, rabbits and guinea pigs, only slight local irritation and no sloughing were induced at the points of injection. The injections were followed by fever of greater or less intensity and duration. The animals appeared to regain their normal condition in varying periods, from four to seven days, but were reserved for future observation. The liquid appeared to be far inferior in its immediate effects when injected subcutaneously, to prussic acid, strychnine and serpent poison; neither did it manifest effects identical with septic poison.

10. Uncoagulated by heat.

11. Uncoagulated by heat and nitric acid.

12. Uncoagulated by nitric acid.

13. Chemically pure, absolute alcohol threw down from the lymph a flocculent whitish deposit.

14. Nitrate of silver threw down a heavy white deposit, showing the presence of chlorides in considerable quantity.

15. Soluble baryta salts gave slight precipitates.

16. Stannous salts gave no evidence of the presence of the salts of gold.

17. Microscopical examination of the undiluted "Koch's lymph," with objectives ranging from 1-5 to 1-15 of an inch, revealed the presence of minute ovoid and rod shaped bodies, resembling the spores and bacillus of the bacillus tuberculosis as originally described by the eminent microscopist, Robert Koch. These micro-organisms in their size structure and behavior, with staining fluids, corresponded with the bacillus tuberculosis.

18 When the "lymph" was diluted with boiled, distilled water and preserved in chemically clean test tubes, the mouths of which were carefully guarded by antiseptic cotton wool, the fluid became turbid. Microscopical examinations revealed the fact, that the turbidity was due to the multiplication of micro-organisms presenting physical and chemical properties similar to those of the bacillus tuberculosis.

19. The addition of a drop of the "lymph" to "Pasteur's sterilized liquid" was followed by the development of the spores and slender, rod-shaped micro-organisms, resembling the bacillus tuberculosis.

20. The spores and bacilli of "Koch's lymph" were cultivated, and with the necessary precautions to exclude all external germs from the atmosphere and internal objects, upon various substances, as serum of blood, boiled potato, white of egg and crystallizable sugar.

21. In cultivations in fresh blood the reaction was strongly alkaline, in those of the potato, white of egg and sugar they were acid.

22. When a small quantity of the lymph was added to a carefully sterilized solution of crystallizable sugar, the clear solution became turbid and emitted a sweetish odor, similar to that which I have often observed to be exhaled by patients suffering from phthisis pulmonalis in the advanced stage.

### CONCLUSIONS.

(*a*) The active principles of "Koch's lymph" appear to reside in a colloid nitrogenized compound, coagulable by absolute alcohol, and in living germs, micro-organisms, spores and bacilli, similar to those of the bacillus tuberculosis, and capable of multiplication within and without the living organism.

(*b*) The potent effects of Koch's lymph, when introduced into the blood of healthy and diseased human beings, may be referred in part at least to the rapid multiplication and action of micro-organisms similar to, if not identical with, the bacillus tuberculosis.

(*c*) The results of the chemical and microscopical examination of the contents of this "vial of Koch's lymph" have led me to exclude this liquid from the list of remedial agents.

I beg to be permitted to say that in the effort to discharge what appeared to be my duty, I have endeavored to serve the art and not the trade of medicine, believing that honorable, legitimate medicine has no secrets to conceal, and holds no remedy which is not the common heritage of the glorious brotherhood of the noble republic of science.

With great respect and with many thanks for the generous consideration of your Excellency, I have the honor to remain

Your obedient servant,

JOSEPH JONES, M. D.,

Professor of Chemistry and Clinical Medicine, Medical Department Tulane University of Louisiana, Visiting Physician Charity Hospital, New Orleans.

